# Optimal Trend Tests for Genetic Association Studies of Heterogeneous Diseases

**DOI:** 10.1038/srep27821

**Published:** 2016-06-09

**Authors:** Wen-Chung Lee

**Affiliations:** 1 Research Center for Genes, Environment and Human Health and Institute of Epidemiology and Preventive Medicine, College of Public Health, National Taiwan University, Taipei, Taiwan

## Abstract

The Cochran-Armitage trend test is a standard procedure in genetic association studies. It is a directed test with high power to detect genetic effects that follow the gene-dosage model. In this paper, the author proposes optimal trend tests for genetic association studies of heterogeneous diseases. Monte-Carlo simulations show that the power gain of the optimal trend tests over the conventional Cochran-Armitage trend test is striking when the genetic effects are heterogeneous. The easy-to-use R 3.1.2 software (R Foundation for Statistical Computing, Vienna, Austria) code is provided. The optimal trend tests are recommended for routine use.

Genetic factors contribute to many human diseases, conferring susceptibility or resistance. Unlike simple Mendelian disorders, more common complex diseases may have many genes involved in their pathogenesis^1^,^2^,^3^. The association of candidate genes (or markers across the genome) with a disease can be efficiently evaluated by a case-control design, in which genotype frequencies are compared for diseased cases and unaffected controls. Genetic association studies are the important first step of gene characterization. Candidate genes or markers found to be statistically significant are then subject to further studies, to identify causal variants, to quantify genetic effects, to examine possible gene-environment or gene-gene interactions, and so on^4^,^5^,^6^,^7^; results from different studies can also be pooled for a meta-analysis^8^,^9^,^10^. The Cochran-Armitage trend test^11^,^12^,^13^,^14^,^15^ has become a standard procedure in this crucial first-step study of complex diseases. It is a directed test most sensitive to detecting genetic effects that follow the gene-dosage model.

However, a disease may comprise more than one disease entity, each with a different etiology, clinical picture and prognosis. Examples of such heterogeneous diseases are Alzheimer’s disease^16^, breast tumors^17^, B-cell lymphoma^18^, acute lymphoblastic leukemia^19^, primary thyroid lymphoma^20^, otosclerosis^21^, rheumatoid arthritis^22^, and autism spectrum disorder^1^. The effect of a gene associated with a heterogeneous disease can be variable, depending on which disease entity one is considering; and if the distinct disease entities themselves, often obscure and subtle, are not recognized and taken into account, the genetic effect associated with the heterogeneous disease at large may vary from person to person.

Genetic heterogeneity can complicate our association study of complex diseases even further. The following hypothetical example should highlight this issue. Consider the disease occurrences in a population of one million people (250,000 people with genotype *aa*; 500,000 people with genotype *Aa*; 250,000 people with genotype *AA*). Assume that the disease under study has two distinct subtypes (which are unknown to researchers). Further assume that both subtypes conform strictly to the gene-dosage model. For Subtype I, the disease risk is 0.0001 for the *aa* genotype, and the risk increases ten-fold per *A* allele; for Subtype II, the disease risk is 0.0020 for the *aa* genotype, and the risk decreases two-fold per *A* allele. A simple calculation shows that the majority (73%) of the diseased subjects in this population are of Subtype I (where the risk increases ten-fold per *A* allele), so the *A* allele should be regarded as a risk allele rather than a protective one. Yet, ignoring the subtypes, we observe disease risks of 0.0021 (*aa* genotype), 0.0020 (*Aa* genotype), and 0.0105 (*AA* genotype), respectively. This is nothing like a gene-dosage model, and moreover, the *A* allele now appears protective, when comparing the *Aa* and the *aa* genotypes. Obviously, applying the standard Cochran-Armitage trend test^11^,^12^,^13^,^14^,^15^ to this setting will result in power loss.

In this paper, we propose optimal trend tests for genetic association studies of heterogeneous diseases.

## Methods

### Notation

For a marker with two alleles *a* and *A*, each individual in a case-control study is genotyped with one of three genotypes, *aa*, *Aa* and *AA* (indexed by *i* = 0, 1, 2, respectively). Assume that the case-control study consists of a total of *n* = *r* + *s* subjects (*r* cases and *s* controls). These *n* subjects can be classified into a 2 × 3 table based on each subject’s genotype and disease status as shown in Table 1.

Let (*x*_0_, *x*_1_, *x*_2_) = (0, *c*, 1) where the coefficient *c* can assume any value. Under the null hypothesis of no genetic association, the following test statistic is distributed asymptotically as a chi-square distribution with one degree of freedom:


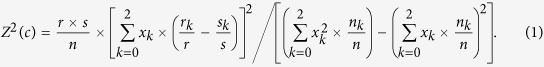


The test with a coefficient of 0.5, *Z*(0.5), is the familiar Cochran-Armitage trend test^11^,^12^,^13^,^14^,^15^.

### Optimal Trend Test

Assume that the non-diseased population is in Hardy-Weinberg equilibrium with an allele frequency (for the *A* allele) of *q*. The expected genotype frequencies for the controls are then, respectively,


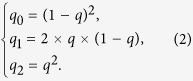


Further assume that the genetic effect is heterogeneous; the allele relative risk (relative risk per *A* allele) is not a constant value but may vary from person to person. Let the expected value of this relative risk be denoted as RR, its coefficient of variation (standard deviation divided by mean; a measure of heterogeneity), as CV_RR_. The expected allele frequency for the cases is then


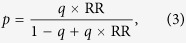


and its variance, calculated by a Taylor approximation (S1 Exhibit), is then


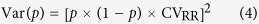


This variance is also the Hardy-Weinberg disequilibrium coefficient in the diseased population, and therefore, the expected genotype frequencies for the cases are, respectively,


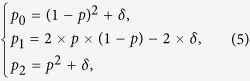


where *δ* = Var(*p*).

In the above calculations, we assumed Hardy-Weinberg equilibrium for the non-diseased population and a gene-dosage genetic model (a constant increase or decrease in risk per *A* allele). We now alleviate these assumptions. In general, the expected genotype frequencies for the controls are, respectively,


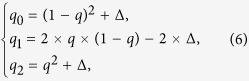


where Δ is the Hardy-Weinberg disequilibrium coefficient in the non-diseased population. The expected genotype relative risks are, respectively,


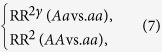


where *γ* is a genetic model parameter. *γ* = 0 corresponds to an autosomal recessive model, *γ* = 0.5, a gene-dosage model, and *γ* = 1, an autosomal dominant model. As before, we allow the parameter RR to have a coefficient of variation CV_RR_, and the parameter *p* (though here it may not be interpreted as the expected allele frequency for the cases) to have a variance as prescribed in Equation (4). Under these conditions, the expected genotype frequencies for the cases (*p*_0_, *p*_1_ and *p*_2_) can be derived from a Taylor expansion. The formulas are rather cumbersome and are relegated to S2 Exhibit.

With the *p*_*i*_ and *q*_*i*_ calculated for *i* = 0, 1 and 2, simple algebra shows that the following optimal coefficient will maximize the test statistic in Equation (1):


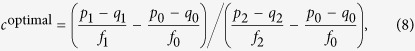


where


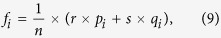


for *i* = 0, 1 and 2, respectively, are the expected genotype frequencies in the total case-control sample. *Z*(*c*^optimal^) is our proposed optimal trend test.

### An Example

We use published case-control data to demonstrate our method. Zhang *et al*.^23^ examined the association between the *adenosine diphosphate ribosyltransferas*e (*ADPRT*) gene (Val762Ala polymorphism) and lung cancer risk. The data (1000 cases and 1018 controls) are shown in Table 2.

For simplicity, we assume Hardy-Weinberg equilibrium for the non-diseased population (with an allele frequency of *q* = 0.4) and a gene-dosage genetic model for the *ADPRT* gene (with a weak association of RR = 1.25 and a moderate heterogeneity of CV_RR_ = 0.4). Using [2]~[5], we then calculate *q*_0_ = (1 − 0.4)^2^ = 0.36, *q*_1_ = 2 × 0.4 × (1 − 0.4) = 0.48, *q*_2_ = 0.4^2^ = 0.16, 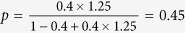, *δ* = Var(*p*) = [0.45 × (1 − 0.45) × 0.4]^2^ = 0.0098, *p*_0_ = (1 − 0.45)^2^ + 0.0098 = 0.31, *p*_1_ = 2 × 0.45 (1 − 0.45) − 2 × 0.0098 = 0.48 and *p*_2_ = 0.45^2^ + 0.0098 = 0.22, respectively.

Using [9], we calculate the expected genotype frequencies in the total case-control sample as 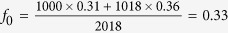, 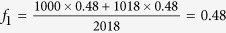, and 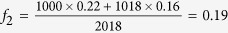, respectively. Using [8], we calculate the optimal coefficient for this example as 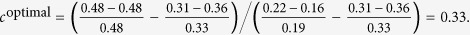


Using [1], we then calculate 
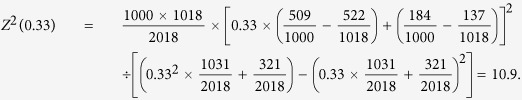


From this, we obtain a very small p-value of 0.00095. By comparison, the conventional Cochran-Armitage trend test for this example results in a higher p-value of 0.00164. Zhang *et al*.^23^ used a chi-square test with two degrees of freedom, which resulted in an even higher p-value of 0.00420. Such differences in p-values should not be taken lightly, considering that a severe multiple-testing penalty often has to be made before declaring significance in a genetic association study.

### Simulation Study

We perform a simulation study to examine the statistical properties of the optimal trend test. The non-diseased population is assumed to be in Hardy-Weinberg equilibrium (Δ = 0), with an allele frequency of *q* = 0.4. We assume a gene-dosage genetic model (*γ* = 0.5), and we consider situations where the *A* allele is a risk allele (RR = 2, 1.5, and 1.25, respectively) and a protective allele (RR = 0.5, 0.67, 0.8, respectively), in turn. For each scenario, we use a sample-size formula for the Cochran-Armitage trend test^13^ to calculate the respective sample size needed for a case-control study (assuming an equal number of cases and controls) to achieve a power of 0.8 at a significance level of 0.05.

We consider various values of CV_RR_: 0.0 (no heterogeneity), 0.1, 0.2,…, 1.0 (profound heterogeneity). For each value of *q*, RRand CV_RR_, we use Equation (8) to calculate the optimal coefficient. We then perform Monte-Carlo simulations (a total of 1,000,000 simulations for each scenario) to calculate the empirical power of the optimal trend test (at the sample sizes described above). For comparison, we also calculate the empirical power of the Cochran-Armitage trend test.

**Figure 1:**
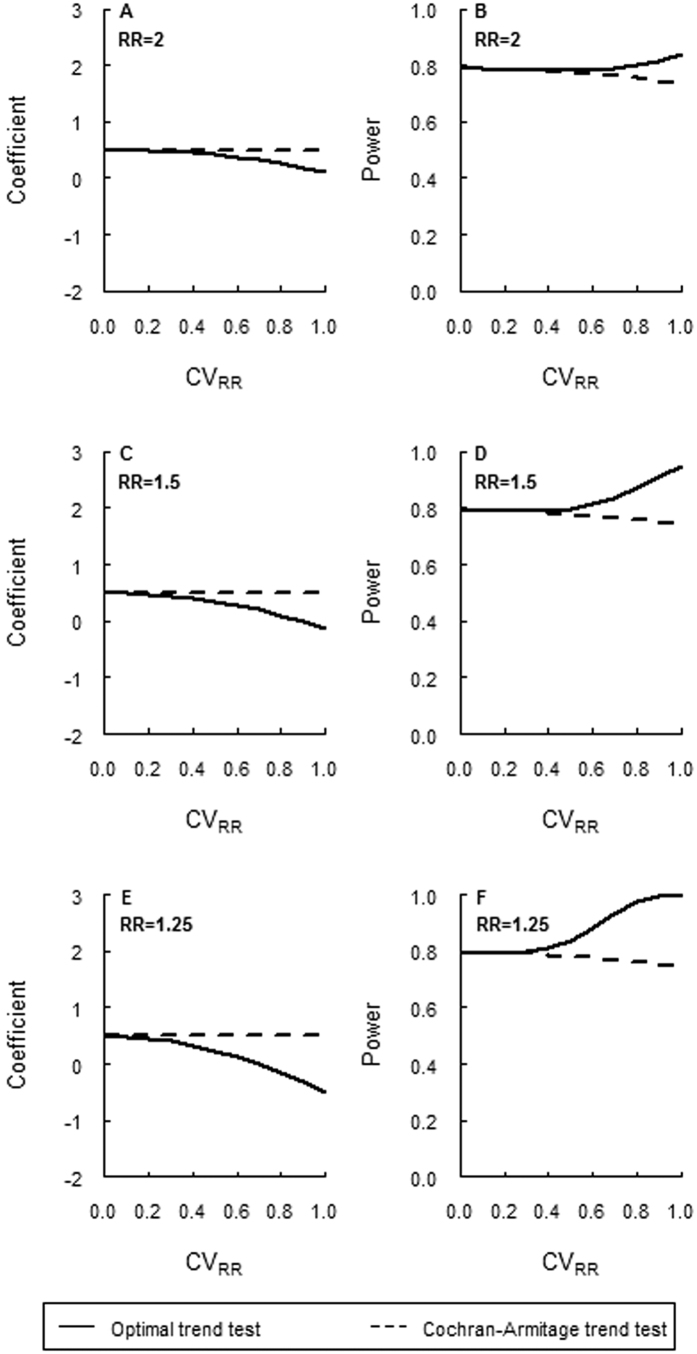
Simulation results for a risk allele ((**A,B**): RR = 2; (**C,D**): RR = 1.5; (**E,F**): RR = 1.25; solid lines: the optimal trend test; dash lines: Cochran-Armitage tend test).

Figure 1 presents the results when the *A* allele is a risk allele (panels A, C, and E for the coefficients; panels B, D and F for the empirical powers). When the genetic effect is homogeneous (CV_RR_ = 0), the optimal coefficients as calculated from Equation (8) are very close to the coefficient of the Cochran-Armitage trend test, namely, 0.5. As a result, the powers of the optimal trend test and the Cochran-Armitage trend test are very similar. As the genetic effect becomes more heterogeneous (larger CV_RR_), the optimal coefficient decreases (down to below zero), and the power of the optimal trend test increases (up to ~100%). The rates of the coefficient decrease/power increase are more striking for a weaker genetic effect (RR = 1.25; panels E and F) than for a stronger genetic effect (RR = 2; panels A and B). By comparison, the Cochran-Armitage trend test uses a constant coefficient of 0.5, and its power decreases gradually with greater heterogeneity.

**Figure 2:**
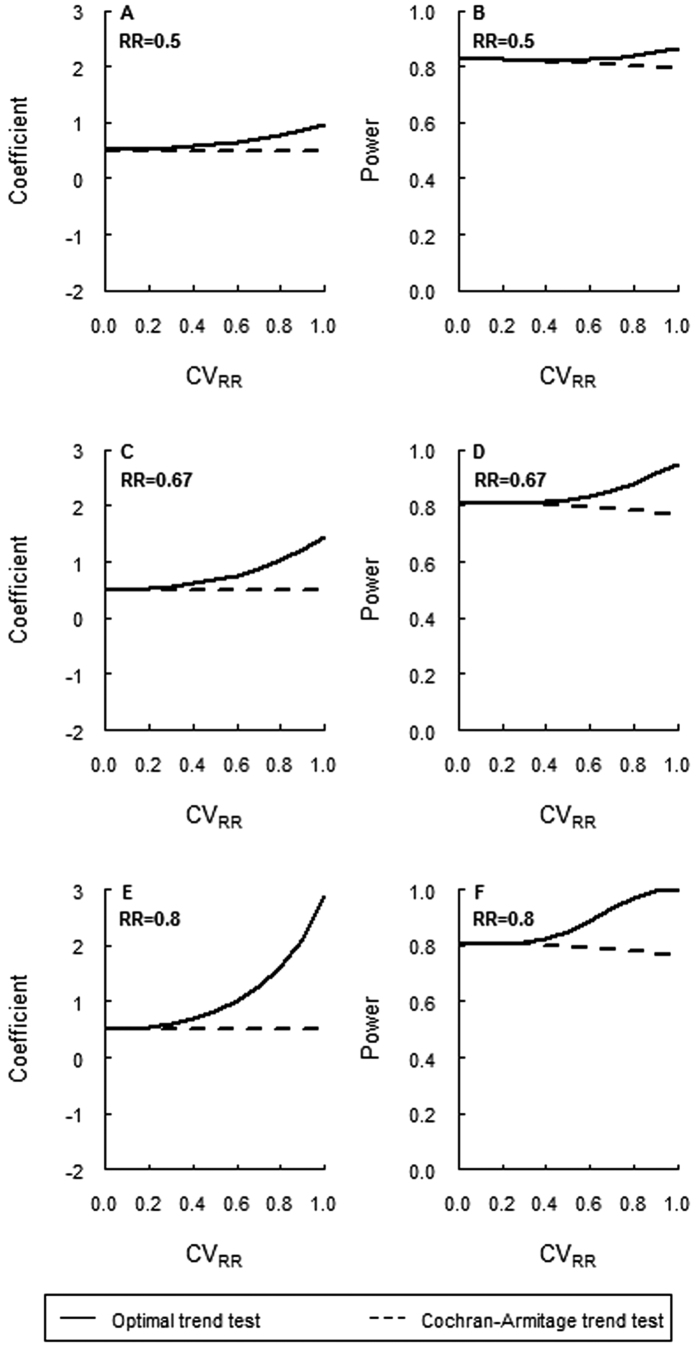
Simulation results for a protective allele ((**A,B**): RR = 0.5; (**C,D**): RR = 0.67; (**E,F**): RR = 0.8; solid lines: the optimal trend test; dash lines: Cochran-Armitage tend test).

Figure 2 presents the results when the *A* allele is a protective allele. Similar findings can be seen in Fig. 1 when *A* is a risk allele, except that as the genetic effect becomes more heterogeneous, the optimal coefficient deviates away from 0.5 in the other direction, increasing up to beyond 1.0 rather than decreasing.

We consider different values of *q*, Δ and*γ*, and the results (S3 Exhibit) all show a superiority of the optimal trend test over the conventional Cochran-Armitage trend test.

## Discussion

The optimal trend test as proposed in this paper is a directed test that is most sensitive for a particular specified alternative. The optimal coefficient depends on the effect of the study gene (mean RR, variability CV_RR_ and genetic model *γ*) and on the underlying population (allele frequency *q*, and Hardy-Weinberg disequilibrium coefficient Δ). This *a priori* information is to be supplied by researchers, either by a literature search or an educated guess. As shown in this study, the power gain over the conventional Cochran-Armitage trend test is striking when the genetic effects are very heterogeneous.

Sometimes, to pinpoint exactly one set of RR, CV_RR_, *γ*, *q* and Δ, calculating the optimal coefficient can be difficult, but suggesting a list of possible sets of parameter values may be easier. Assuming that a researcher comes up with a total of *m* sets of parameter values, he/she can input these into our Equation (8) to calculate a total of *m* optimal coefficients, 
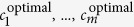
 and then input these into our Equation (1) for a total of *m* optimal trend tests. Next, a summary test can be performed based on a weighted sum of these *m* test statistics:


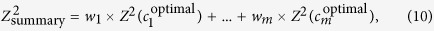


where *w*_1_, …, *w*_*m*_ are the weights given to reflect the plausibility of each set of parameter values. The multiple testing problem should not concern us here, because we make one and only one summary test. Under the null hypothesis of no genetic association, 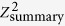 is distributed asymptotically as a mixture of chi-square variables (detailed in S4 Exhibit). (The test reduces to the optimal trend test in this paper when *m* = 1)

The proposed optimal trend tests (and the summary test) are easy to calculate. S5 Exhibit presents the R 3.1.2 software (R Foundation for Statistical Computing, Vienna, Austria) code and a number of worked examples. The R program also allows for the direct input of the optimal coefficients. For example, if one suspects a gene-dosage model with heterogeneous effects, one can input one coefficient slightly above 0.5, say *c*_1_ = 0.8, another coefficient slightly below 0.5, say *c*_2_ = 0.2 and *w*_1_ = *w*_2_ = 1, to the R program to test 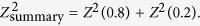 As another example, if one is uncertain about the genetic model, one can input *c*_1_ = 0.5 (gene dosage), *c*_2_ = 1 (autosomal dominant), *c*_3_ = 0 (autosomal recessive), and *w*_1_ = *w*_2_ = *w*_3_ = 1 into the R program to test 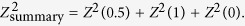


## Additional Information

**How to cite this article**: Lee, W.-C. Optimal Trend Tests for Genetic Association Studies of Heterogeneous Diseases. *Sci. Rep*. **6**, 27821; doi: 10.1038/srep27821 (2016).

## Supplementary Material

Supplementary Information

## Figures and Tables

**Table 1:** Genotype distribution for case-control studies.

	*aa*	*Aa*	*AA*	Total
Cases	*r*_0_	*r*_1_	*r*_2_	*r*
Controls	*s*_0_	*s*_1_	*s*_2_	*s*
Total	*n*_0_	*n*_1_	*n*_2_	*n*

**Table 2:** Association between the *adenosine diphosphate ribosyltransferas*e (*ADPRT*) gene (Val762Ala polymorphism) and lung cancer risk (data taken from ref. 23).

	Val/Val	Val/Ala	Ala/Ala	Total
Cases	307	509	184	1000
Controls	359	522	137	1018
Total	666	1031	321	2018
