# Supplementary material for: Optimal Trend Tests for Genetic Association Studies of Heterogeneous Diseases
Source: Sci Rep. 2016 Jun 9;6:27821. doi: 10.1038/srep27821 (PMC4899796; doi:10.1038/srep27821)
Supplement: Supplementary Information [file srep27821-s1.pdf]

Title: Optimal Trend Tests for Genetic Association Studies of Heterogeneous Diseases

Author: Wen-Chung Lee

Supplementary information:

S1 Exhibit. A Taylor approximation for the variance of the allele frequency for the cases.

S2 Exhibit. Formulas for the expected genotype frequencies under general conditions.

S3 Exhibit. Simulation results using different parameter values.

S4 Exhibit. The summary test.

S5 Exhibit. R code and a number of worked examples.

S1 Exhibit. A Taylor approximation for the variance of the allele frequency for the cases.

$$
\begin{aligned}
\mathrm{Var}(p) &= \mathrm{Var}\left(\frac{q\times \mathrm{RR}}{1-q+q\times \mathrm{RR}}\right) \\
&= \left[\frac{d}{d\mathrm{RR}}\left(\frac{q\times \mathrm{RR}}{1-q+q\times \mathrm{RR}}\right)\right]^2 \times \mathrm{Var}(\mathrm{RR}) \\
&= \left[\frac{q\times(1-q)}{\left(1-q+q\times \mathrm{RR}\right)^2}\right]^2 \times \mathrm{RR}^2 \times \mathrm{CV}_{\mathrm{RR}}^2 \\
&= \left[\frac{q\times \mathrm{RR}}{1-q+q\times \mathrm{RR}} \times \frac{1-q}{1-q+q\times \mathrm{RR}}\right]^2 \times \mathrm{CV}_{\mathrm{RR}}^2 \\
&= \left[p\times(1-p)\times \mathrm{CV}_{\mathrm{RR}}\right]^2 .
\end{aligned}
$$

S2 Exhibit. Formulas for the expected genotype frequencies under general conditions.

From the function $p = \frac{q \times \mathrm{RR}}{1 - q + q \times \mathrm{RR}}$ defined in the text, we first calculate the following derivatives:

$$\frac{d\mathrm{RR}}{dp} = (1-p)^{-2} \times \left(\frac{q}{1-q}\right)^{-1}$$

and

$$\frac{d^2\mathrm{RR}}{dp^2} = 2 \times (1-p)^{-3} \times \left(\frac{q}{1-q}\right)^{-1}.$$

Next, let $F_0 = q_0$, $F_1 = q_1 \times \mathrm{RR}^{2\gamma}$ and $F_2 = q_2 \times \mathrm{RR}^2$, where $q_0 = (1-q)^2 + \Delta$, $q_1 = 2 \times q \times (1-q) - 2 \times \Delta$ and $q_2 = q^2 + \Delta$, respectively, and let $F_+ = \sum_{j=0}^{2} F_j$. The derivatives of these functions with respect to p are

$$\frac{dF_0}{dp} = 0,$$
$$\frac{dF_1}{dp} = q_1 \times (2\gamma) \times \mathrm{RR}^{2\gamma-1} \times \frac{d\mathrm{RR}}{dp},$$
$$\frac{dF_2}{dp} = q_2 \times 2 \times \mathrm{RR} \times \frac{d\mathrm{RR}}{dp},$$

$$\frac{d^2F_0}{dp^2} = 0,$$

$$\frac{d^2F_1}{dp^2} = q_1 \times (2\gamma) \times (2\gamma - 1) \times \mathrm{RR}^{2\gamma-2} \times \left(\frac{d\mathrm{RR}}{dp}\right)^2 + q_1 \times (2\gamma) \times \mathrm{RR}^{2\gamma-1} \times \frac{d^2\mathrm{RR}}{dp^2},$$

$$\frac{d^2F_2}{dp^2} = q_1 \times 2 \times \left(\frac{d\mathrm{RR}}{dp}\right)^2 + q_1 \times 2 \times \mathrm{RR} \times \frac{d^2\mathrm{RR}}{dp^2},$$

$$\frac{dF_+}{dp} = \sum_{j=0}^{2} \frac{dF_j}{dp},$$

and

$$\frac{d^2F_+}{dp^2} = \sum_{j=0}^{2} \frac{d^2F_j}{dp^2},$$

respectively. Let $R_j = \frac{F_j}{F_+}$ for $j = 0$, 1, and 2. The second derivatives of these ratio functions are

$$\frac{d^2R_j}{dp^2} = \frac{d^2F_j}{dp^2} \times F_+^{-1} - F_j \times \frac{d^2F_+}{dp^2} \times F_+^{-2}$$
$$- 2 \times \frac{dF_j}{dp} \times \frac{dF_+}{dp} \times F_+^{-2} + 2 \times F_j \times \left(\frac{dF_+}{dp} \right)^2 \times F_+^{-3},$$

for $j = 0$, 1, and 2. The expected genotype frequencies for the cases are then

$$p_j = R_j + \frac{1}{2} \times \frac{d^2R_j}{dp^2} \times \mathrm{Var}(p),$$

for $j = 0$, 1, and 2, where $\mathrm{Var}(p) = \left[p \times (1-p) \times \mathrm{CV_{RR}} \right]^2$ as prescribed in the text.

S3 Exhibit. Simulation results using different parameter values.

$q = 0.2$, $\Delta = 0$ and $\gamma = 0.5$

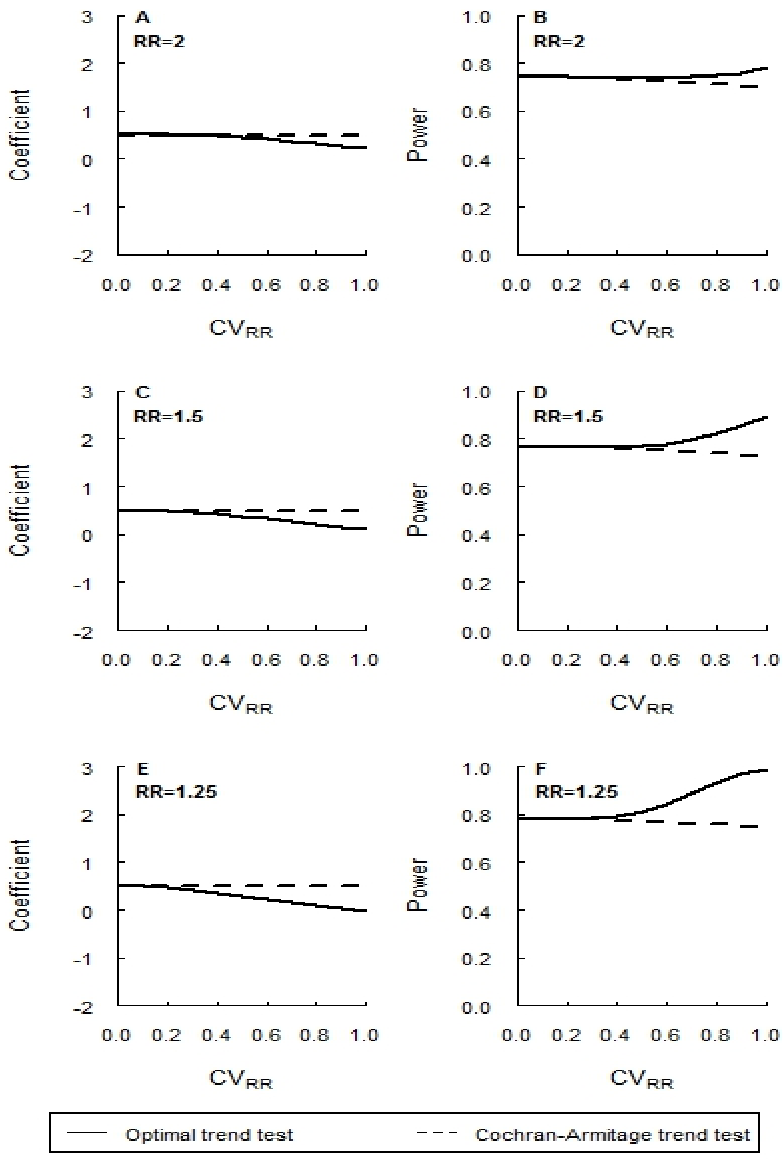

$q = 0.2$, $\Delta = 0$ and $\gamma = 0.5$

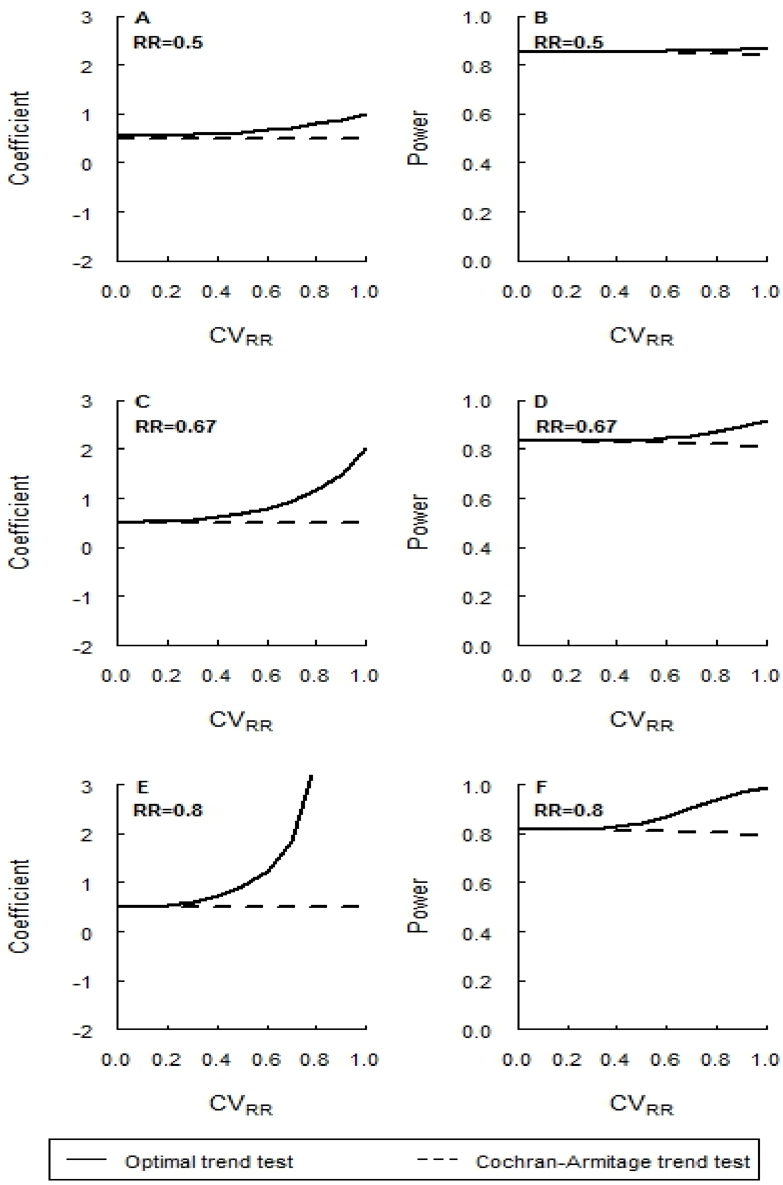

$q = 0.8$, $\Delta = 0$ and $\gamma = 0.5$

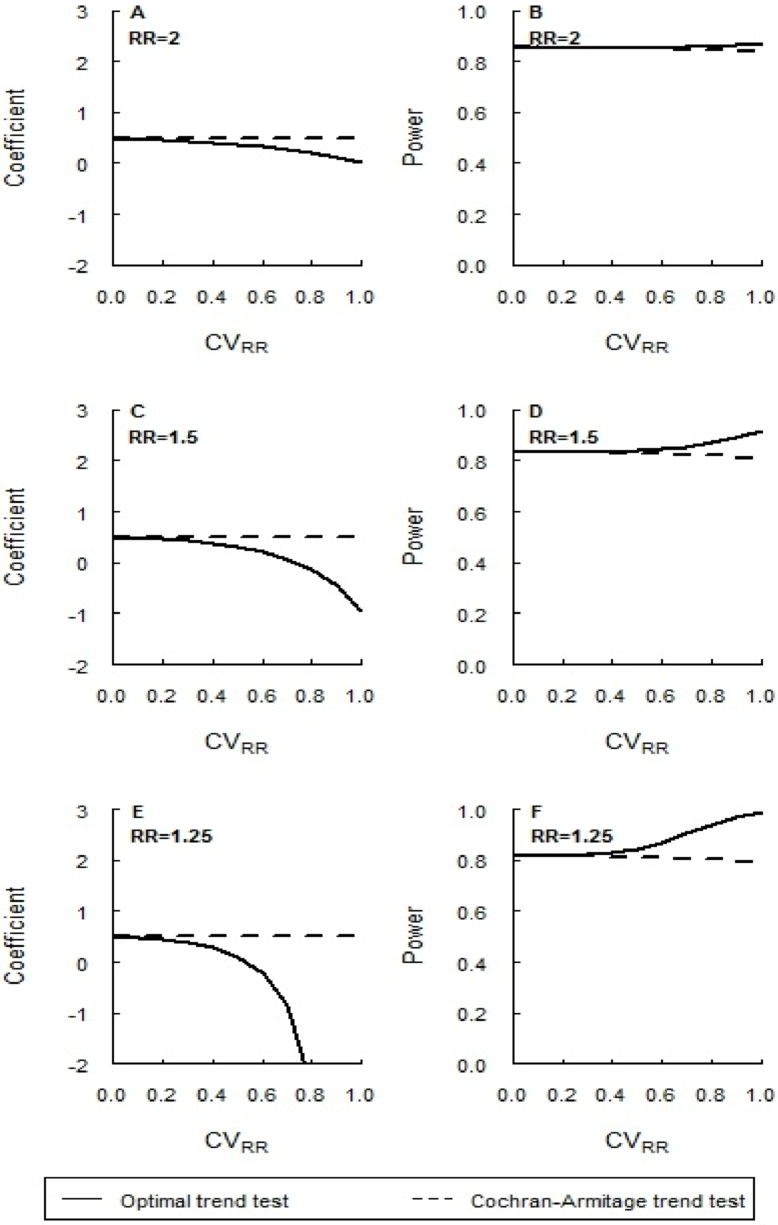

$q = 0.8$, $\Delta = 0$ and $\gamma = 0.5$

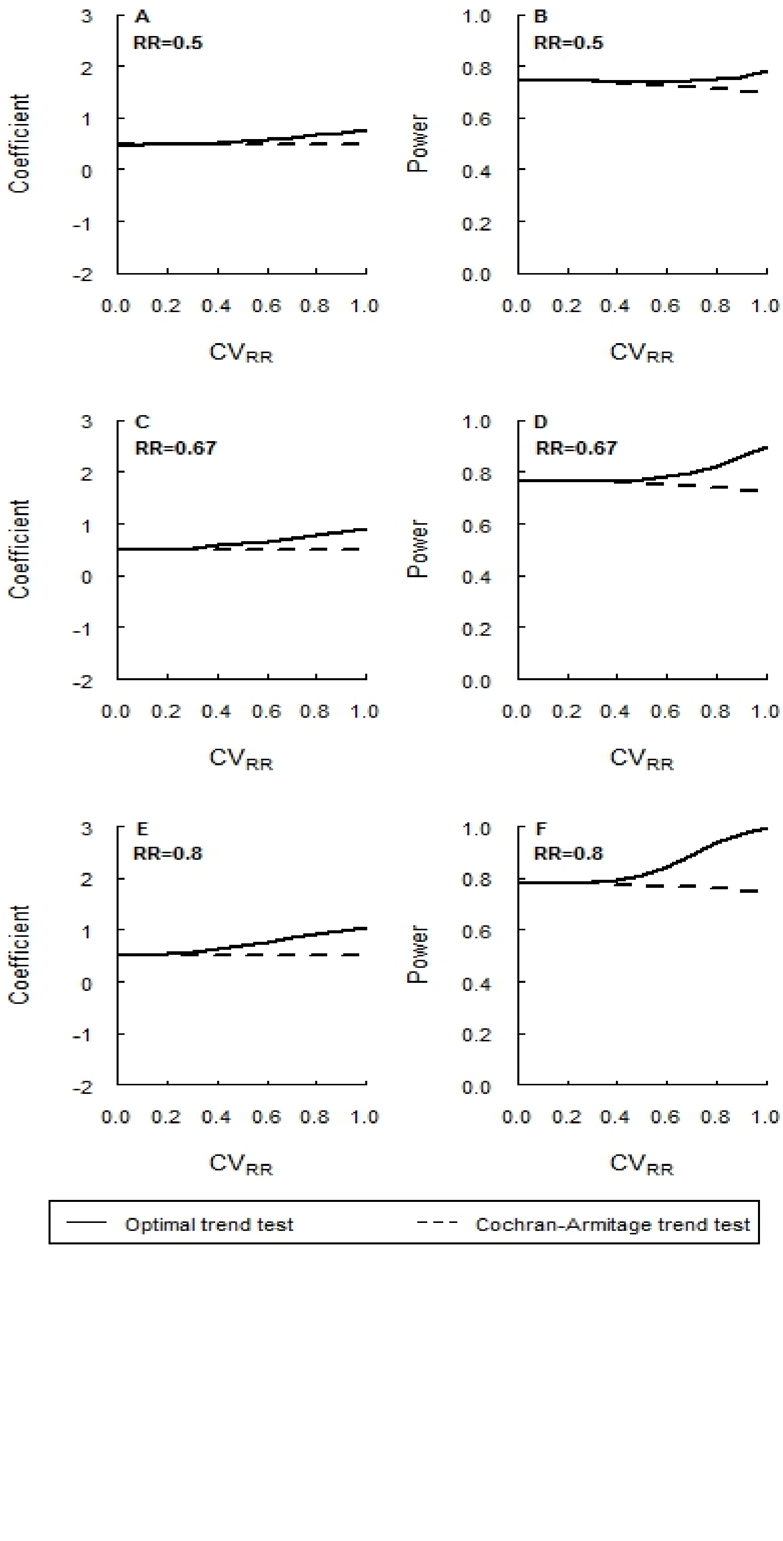

$q = 0.4$, $\Delta = -0.005$ and $\gamma = 0.5$

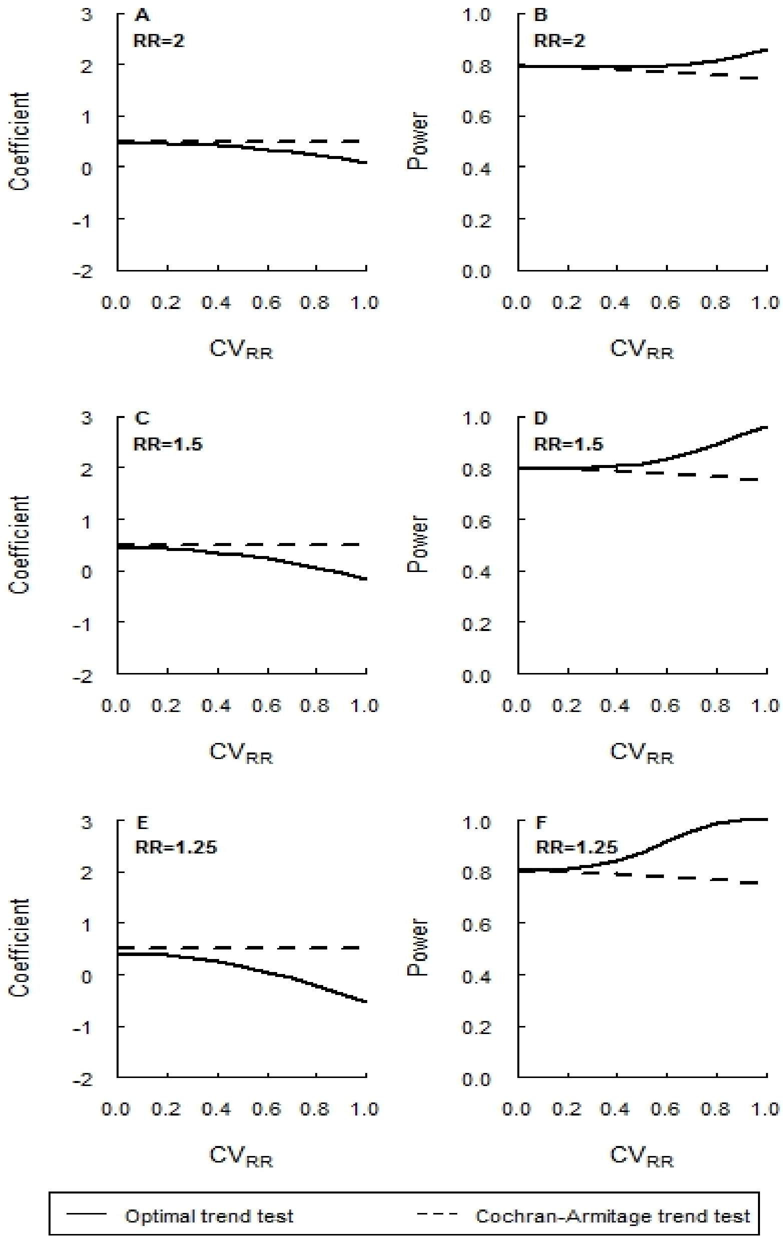

$q = 0.4$, $\Delta = -0.005$ and $\gamma = 0.5$

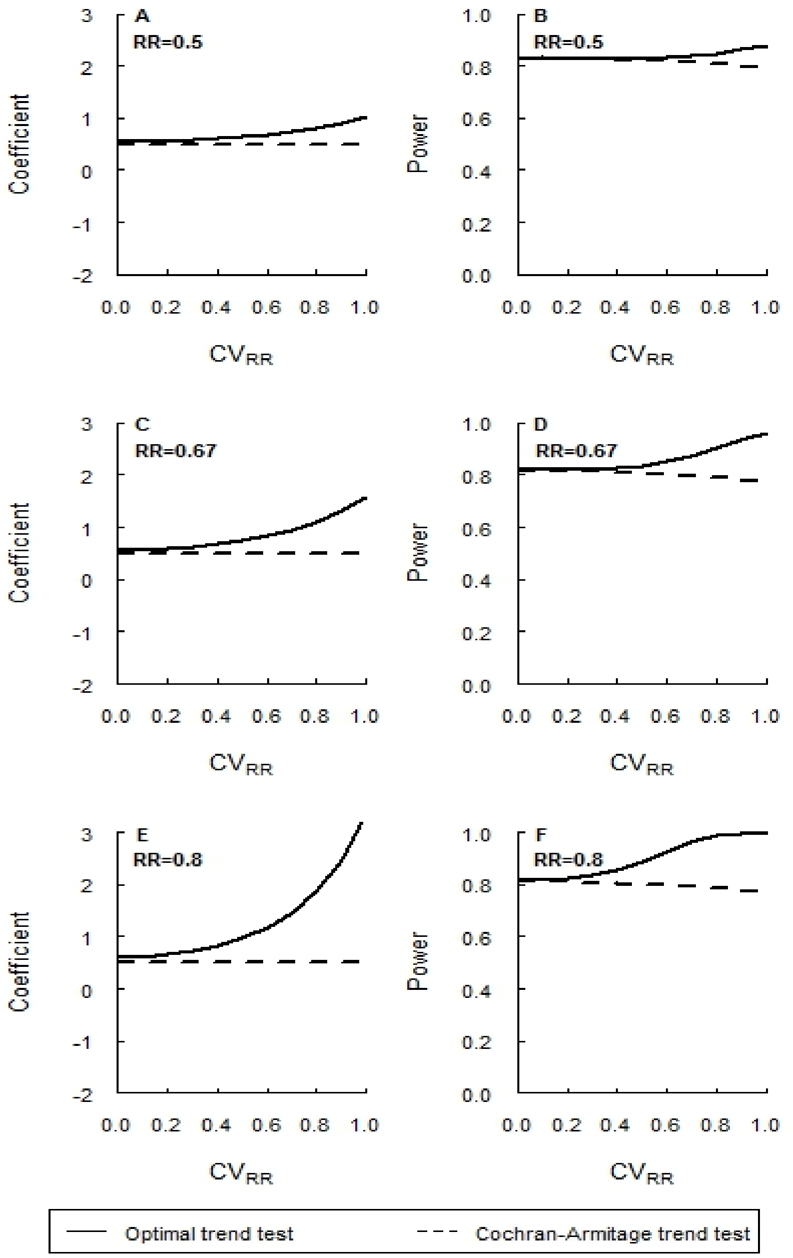

$q = 0.4$, $\Delta = 0.005$ and $\gamma = 0.5$

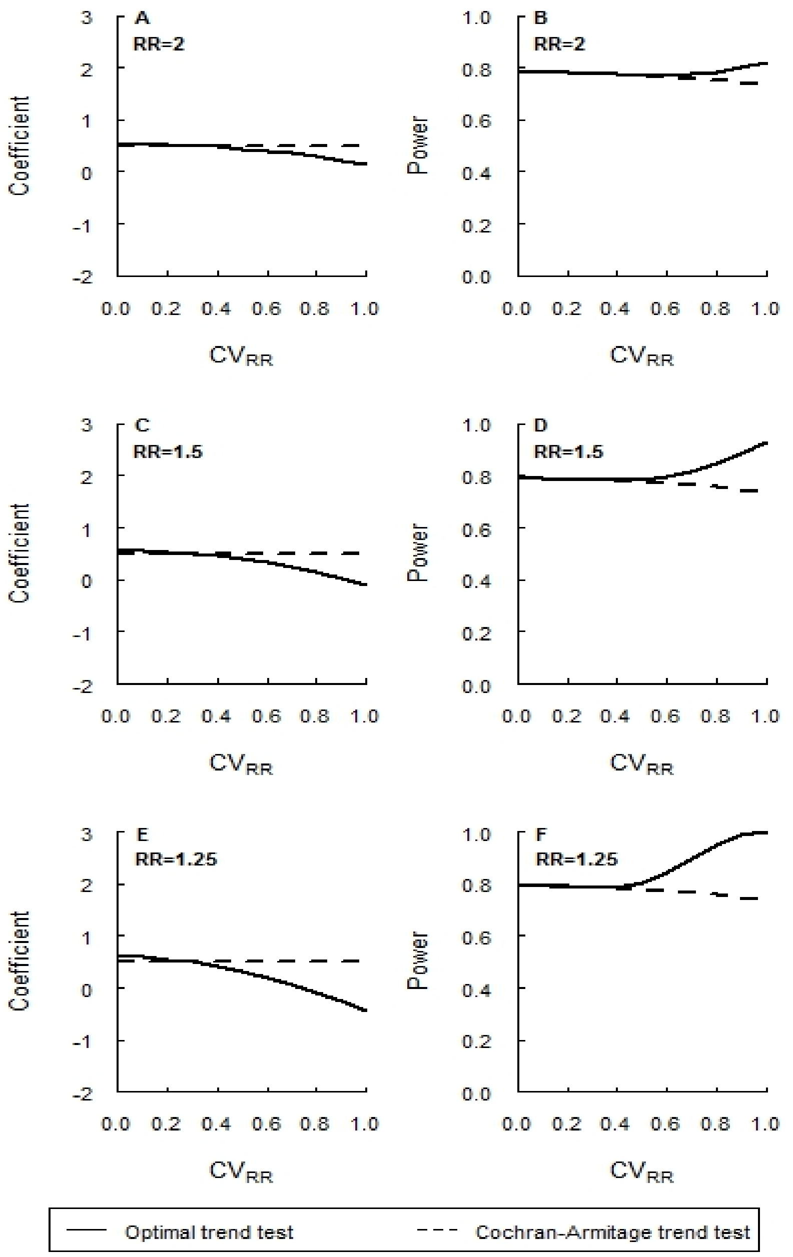

$q = 0.4$, $\Delta = 0.005$ and $\gamma = 0.5$

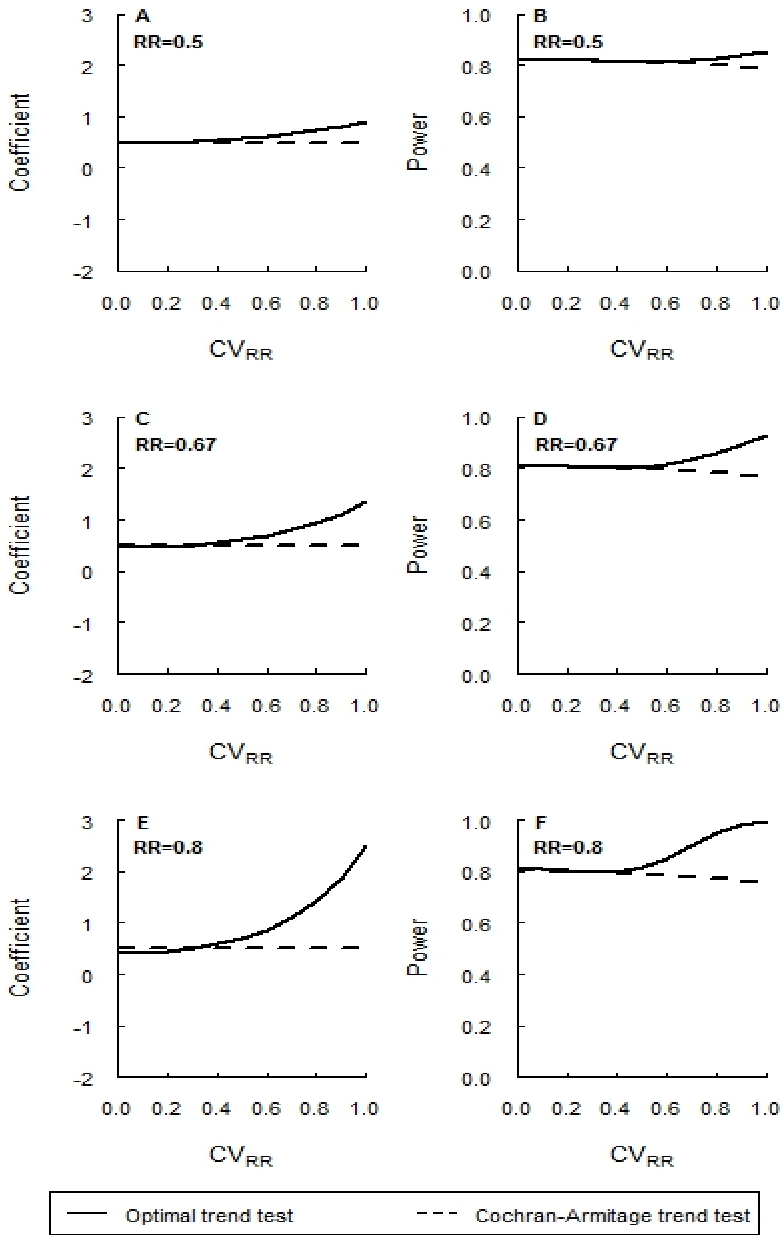

$q = 0.4$, $\Delta = 0$ and $\gamma = 0.3$

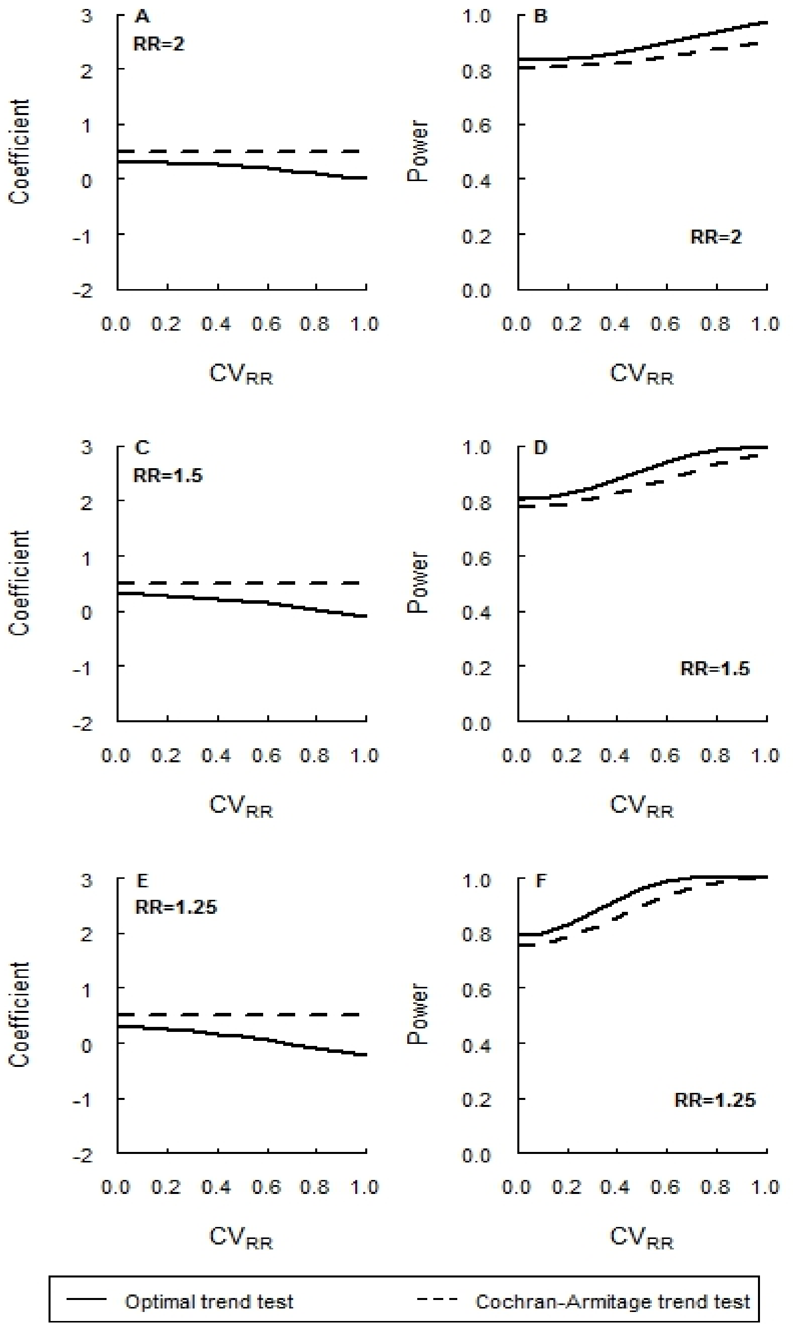

$q = 0.4$, $\Delta = 0$ and $\gamma = 0.3$

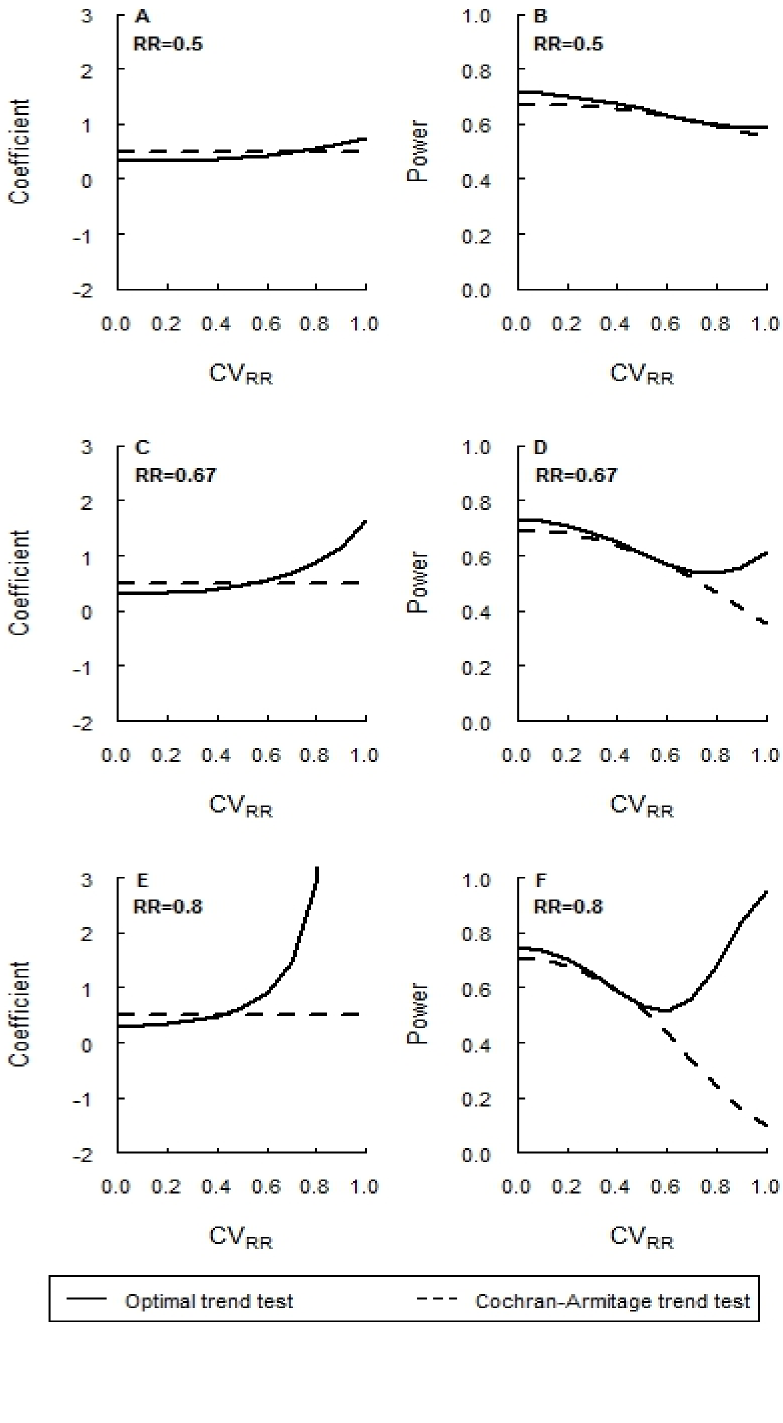

$q = 0.4$, $\Delta = 0$ and $\gamma = 0.7$

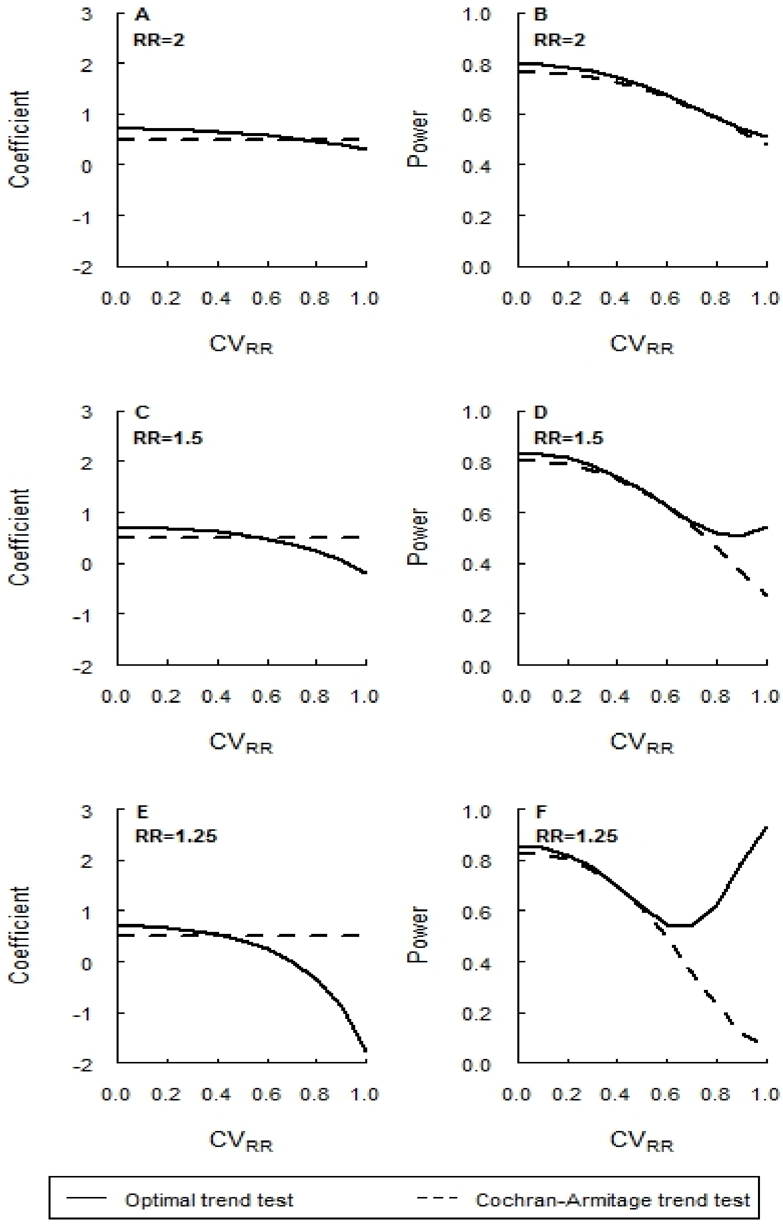

$q = 0.4$, $\Delta = 0$ and $\gamma = 0.7$

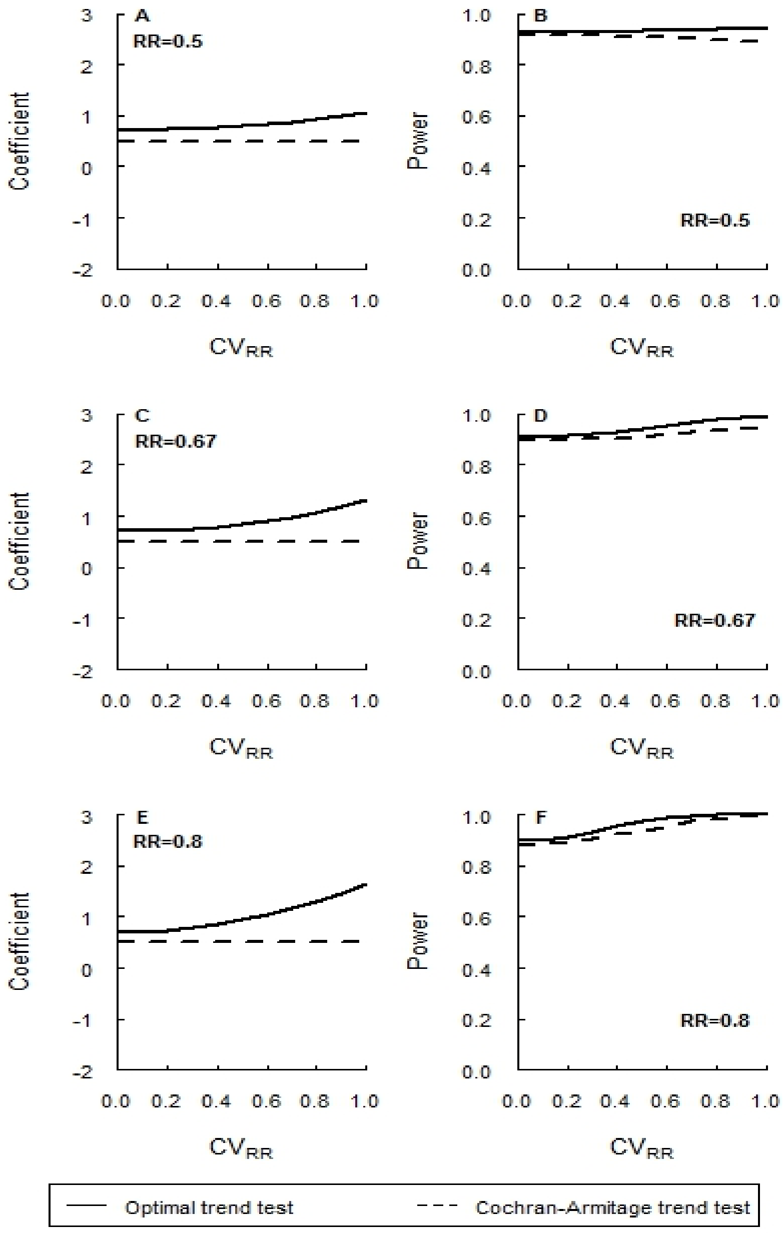

S4 Exhibit. The summary test.

Let the disease statuses (coded 1 for cases, and 0, for controls) of the study subjects be represented in $\mathbf{y}$, an $n \times 1$ vector, with $\mathbf{y}^{\mathrm{t}} = \left[\mathbf{1}_{r_0}^{\mathrm{t}},\ \mathbf{0}_{s_0}^{\mathrm{t}},\ \mathbf{1}_{r_1}^{\mathrm{t}},\ \mathbf{0}_{s_1,}^{\mathrm{t}}\ \mathbf{1}_{r_2}^{\mathrm{t}},\ \mathbf{0}_{s_2}^{\mathrm{t}}\right]$ where $\mathbf{1}_a$ and $\mathbf{0}_b$ are $a \times 1$ vector of 1's and $b \times 1$ vector of 0's, respectively. Let the genotype coefficients be represented in $\mathbf{X}$, an $n \times m$ matrix, with its j th column $\mathbf{x}_j$ being $\mathbf{x}_j^{\mathrm{t}} = \left[\mathbf{0}_{n_0}^{\mathrm{t}},\ c_j^{\text{optimal}} \times \mathbf{1}_{n_1}^{\mathrm{t}},\ \mathbf{1}_{n_2}^{\mathrm{t}}\right]$. We standardize $\mathbf{y}$ and the columns of $\mathbf{X}$ to have zero means and unit variances: $\tilde{\mathbf{y}} = \left(\mathbf{y} - \frac{r}{n}\right) \Big/ \sqrt{\frac{r}{n} \times \frac{s}{n}}$ and $\tilde{\mathbf{X}} = \left(\mathbf{X} - \mathbf{1}_n \overline{\mathbf{x}}\right)\mathbf{D}^{-1/2}$, respectively, where $\overline{\mathbf{x}} = \frac{1}{n} 1_n^{\mathrm{t}} \mathbf{X}$ is a $1 \times m$ vector of the column means of $\mathbf{X}$, and $\mathbf{D}^{1/2}$ is an $m \times m$ diagonal matrix with the column standard deviations of $\mathbf{X}$ in its diagonal. [The diagonal of $\mathbf{D}^{1/2}$ is the square root of the diagonal of the variance-covariance matrix, $\mathbf{S} = \frac{1}{n}\mathbf{X}^{\mathrm{t}}\mathbf{X} - \overline{\mathbf{x}}^{\mathrm{t}}\overline{\mathbf{x}}$.] Next, we calculate an $n \times n$ kernel matrix: $\mathbf{K} = \frac{1}{n}\tilde{\mathbf{X}}\mathrm{Diag}(\mathbf{w})\tilde{\mathbf{X}}^{\mathrm{t}}$, where $\mathrm{Diag}(\mathbf{w})$ is an $m \times m$ diagonal matrix with the weights, $w_1, \ldots, w_m$, in its diagonal.

In matrix notation, the summary test statistic described in the text is a quadratic from: $Z_{\text{summary}}^2 = \tilde{\mathbf{y}}^{\mathrm{t}}\mathbf{K}\tilde{\mathbf{y}}$. Under the null hypothesis of no genetic association, this quadratic from is asymptotically distributed as a mixture of chi-squared variables: $\sum_{i=1}^{n} \lambda_i \times \chi_{\mathrm{df}=1,i}^2$, where $\chi_{\mathrm{df}=1,i}^2$'s are independent $\mathrm{df} = 1$ chi-squared variables and $\lambda_1 \geq \lambda_2 \geq \ldots \geq \lambda_n$ are the ordered eigenvalues of $\mathbf{K}$. (For the 2×3 table of case-control data, there are at most two eigenvalues that are large than zero, and all the rest are zeros.) This mixture of chi-squares can be approximated by the three-moment

approximation method, and the P-value is given by $\text{P-value} = \Pr\left[\chi^2_{\text{df}=b} > \left(Z^2_{\text{summary}} - a_1 \right) \times \sqrt{\frac{b}{a_2}} + b \right]$,

where $a_u = \sum_{i=1}^{n} \lambda_i^u$, $b = \frac{a_2^3}{a_3^2}$, and $\chi^2_{\text{df}=b}$ is the $\text{df} = b$ chi-squared distribution.

S5 Exhibit. R code and a number of worked examples.

```
########################################################
#   Arguments for the function, optimal.trend.test:                #
#       case: the numbers of cases (a vector of length 3)          #
#       control: the numbers of controls (a vector of length 3)    #
#       RR: mean genetic effect(s)                                 #
#       cv.RR: coefficient(s) of variation of the genetic effects  #
#       gamma: genetic model parameter(s) (default=0.5)            #
#       q: allele frequenc(ies) (default=0.5)                      #
#       HWD: Hardy-Weinberg disequilibrium coefficient(s)          #
#               in the non-diseased population (default=0)         #
#       coef.opt: directly inputting the optimal coefficient(s)    #
#                   (default=NULL)                                 #
#       w: weight(s) (default=1)                                   #
########################################################
optimal.trend.test=function( case, control,
                             RR, cv.RR, gamma=0.5,
                             q=0.5, HWD=0,
                             coef.opt=NULL,
                             w=1 ){
  N1=sum(case)
  N0=sum(control)
  NT=N1+N0
  total=case+control
  tot.prop=total/NT
  cscndiff.vector=case/N1 - control/N0
  mean.y=N1/NT
  var.y= N1*N0/NT/NT
  y.vector=matrix(
          c( rep(1,case[1]), rep(0,control[1]),
             rep(1,case[2]), rep(0,control[2]),
             rep(1,case[3]), rep(0,control[3]) ),
        NT,1)
  y.stand=(y.vector-mean.y)/sqrt(var.y)
  len=length(coef.opt)
```

```
if (len==0){
   len=length(cv.RR)*length(RR)*length(q)*
         length(HWD)*length(gamma)*length(w)
   cv.RR=rep(cv.RR, length=len)
   RR=rep(RR, length=len)
   q=rep(q, length=len)
   HWD=rep(HWD, length=len)
   gamma=rep(gamma, length=len)
   coef.opt=1:len
   for (j in 1:len){
        fcn= c( (1-q[j])^2, 2*q[j]*(1-q[j]), q[j]^2) +
                  c(HWD[j], -2*HWD[j], HWD[j])
        p=q[j]*RR[j] / ( 1-q[j] + q[j]*RR[j])
        delta=( p*(1-p)*cv.RR[j] )^2
        odds=q[j]/(1-q[j])
        d.RR= (1-p)^(-2) /odds
        dd.RR= 2 *(1-p)^(-3) /odds
        F=fcn*c(1, RR[j]^(2*gamma[j]), RR[j]^2)
        d.F= fcn*c( 0,
                       2*gamma[j]* RR[j] ^( 2*gamma[j] - 1) * d.RR,
                       2*RR[j] * d.RR )
        dd.F= fcn*c( 0,
                        2*gamma[j]*( 2*gamma[j] - 1)*
                             RR[j] ^( 2*gamma[j] - 2) * d.RR^2 +
                             2*gamma[j]* RR[j] ^( 2*gamma[j] - 1) * dd.RR,
                        2* d.RR^2 + 2*RR[j] * dd.RR )
        G=sum(F)
        d.G=sum(d.F)
        dd.G=sum(dd.F)
        dd.F_G=dd.F/ G - F*dd.G/ G^2 -
                    2*d.F*d.G/ G^2 + 2*F*d.G^2/ G^3
        fcs=F/G + dd.F_G/2 * delta
        freq=(N1*fcs + N0*fcn)/NT
        dif=fcs-fcn
        coef.opt[j]= ( dif[2]/freq[2] - dif[1]/freq[1] ) /
                        ( dif[3]/freq[3] - dif[1]/freq[1] )
   }
}
```

```
w=rep(w, length=len)
w=w/sum(w)
stat.optimal=1:len
x.stand=matrix(0,NT,len)
for (j in 1:len){
    coefx=c(0, coef.opt[j], 1)
    coefx.sq=coefx^2
    mean.x=crossprod(coefx, tot.prop)
    var.x=crossprod(coefx.sq, tot.prop) - mean.x ^2
    cscndiff=crossprod(coefx, cscndiff.vector)
    stat.optimal[j]=N1*N0/NT* cscndiff^2 / var.x
    coefx.stand=(coefx-mean.x)/sqrt(var.x)
    x.stand[,j]=matrix(
                    c( rep(coefx.stand[1], total[1]),
                       rep(coefx.stand[2], total[2]),
                       rep(coefx.stand[3], total[3]) ),
                   NT, 1)
}
Ker=x.stand %*% diag(w) %*% t(x.stand) / NT
Qform=t(y.stand) %*% Ker %*% y.stand
lambda=eigen(Ker)$values[1:2]
moment.1=sum(lambda)
moment.2=sum(lambda^2)
moment.3=sum(lambda^3)
freedom=moment.2^3 / moment.3^2
p.value.optimal=1- pchisq(stat.optimal, df=1)
p.value.mix=1- pchisq( (Qform-moment.1)*
                        sqrt(freedom/moment.2) + freedom,
                        df=freedom )
coef5= c(0,0.5,1)
coef5.sq=coef5^2
mean.5=crossprod(coef5, tot.prop)
var.5=crossprod(coef5.sq, tot.prop) - mean.5 ^2
cscndiff=crossprod(coef5, cscndiff.vector)
stat.Armitage=N1*N0/NT* cscndiff^2 / var.5
p.value.Armitage=1- pchisq(stat.Armitage, df=1)
RESULTS=list( Optimal.Coefficients=coef.opt,
              Optimal.Trend.Tests=p.value.optimal,
```

```
                Summary.Test=p.value.mix,
                Cochran.Armitage.Trend.Test=p.value.Armitage )
  return(RESULTS)
}

##################################################
#                  Worked Examples                   #
##################################################
# case-control data
  case=c(98, 167, 135)      # cases
  control=c(100, 200, 100)      # controls

# Example 1
  optimal.trend.test( case=case, control=control,    # the data
                      RR=1.2,      # mean genetic effect
                      cv.RR=0.8      # heterogeneity
                     )

# Example 2
  optimal.trend.test( case=case, control=control,    # the data
                      RR=c(1.2, 1/1.2),      # risk or protective ??
                      cv.RR=0.8      # heterogeneity
                     )

# Example 3
  optimal.trend.test( case=case, control=control,    # the data
                      RR=seq(1.1, 3.0, by=0.1), #    magnitude of risk ??
                      cv.RR=0.8      # heterogeneity
                     )

# Example 4
  optimal.trend.test( case=case, control=control,    # the data
                      RR=1.2,      # mean genetic effect
                      cv.RR=0.8,      # heterogeneity
                      q=seq(0.1, 0.9, by=0.1)       # allele frequency ??
                     )

# Example 5
```

```
   optimal.trend.test( case=case, control=control,    # the data
                       RR=1.2,      # mean genetic effect
                       cv.RR=seq(0.0, 1.0, by=0.1)    # heterogeneity ??
                      )

# Example 6
   optimal.trend.test( case=case, control=control,    # the data
                       RR=seq(1.1, 2.0, by=0.1), #    magnitude of risk ??
                       cv.RR=seq(0.0, 1.0, by=0.1),    # heterogeneity ??
                       q=seq(0.1, 0.9, by=0.1)        # allele frequency ??
                      )

# Example 7 (directly inputting the coefficients; effect heterogeneity)
   optimal.trend.test( case=case, control=control,    # the data
                       coef.opt=c(0.8, 0.2)      # likely coefficients
                      )

# Example 8 (directly inputting the coefficients; model uncertainty)
   optimal.trend.test( case=case, control=control,    # the data
                       coef.opt=c(0.5, 1, 0)    # likely coefficients
                      )
```
